# The impact of frailty and sarcopenia on postoperative outcomes in older patients undergoing gastrectomy surgery: a systematic review and meta-analysis

**DOI:** 10.1186/s12877-017-0569-2

**Published:** 2017-08-21

**Authors:** Yanjiao Shen, Qiukui Hao, Jianghua Zhou, Birong Dong

**Affiliations:** 1 The Center of Gerontology and Geriatrics, West China Hospital, Sichuan University, #37 Guoxuexiang, Chengdu, Sichuan 610041 China; 2 The First Affiliated Hospital Medical College, Chengdu Medical College, No. 278 Middle of Baoguang Road, Xindu District, Chengdu, Sichuan 610500 China; 3 Collaborative Innovation Center of Sichuan for Elderly Care and Health, No. 783, Xindu Lu, Chengdu, Sichuan 610500 China

**Keywords:** Gastric cancer, Sarcopenia, Frailty, Postoperative complications

## Abstract

**Background:**

Gastric cancer is a major health problem, and frailty and sarcopenia will affect the postoperative outcomes in older people. However, there is still no systematic review to determine the role of frailty and sarcopenia in predicting postoperative outcomes among older patients with gastric cancer who undergo gastrectomy surgery.

**Methods:**

We searched Embase, Medline through the Ovid interface and PubMed websites to identify potential studies. All the search strategies were run on August 24, 2016. We searched the Google website for unpublished studies on June 1, 2017. The data related to the endpoints of gastrectomy surgery were extracted. Odds ratios (ORs) and their 95% confidence intervals (CIs) were pooled to estimate the association between sarcopenia and adverse postoperative outcomes by using Stata version 11.0. PRISMA guidelines for systematic reviews were followed.

**Results:**

After screening 500 records, we identified eight studies, including three prospective cohort studies and five retrospective cohort studies. Only one study described frailty, and the remaining seven studies described sarcopenia. Frailty was statistically significant for predicting hospital mortality (OR 3.96; 95% CI: 1.12–14.09, *P* = 0.03). Sarcopenia was also associated with postoperative outcomes (pooled OR 3.12; 95% CI: 2.23–4.37). No significant heterogeneity was observed across these pooled studies (Chi^2^ = 3.10, I^2^ = 0%, *P* = 0.685).

**Conclusion:**

Sarcopenia and frailty seem to have significant adverse impacts on the occurrence of postoperative outcomes. Well-designed prospective cohort studies focusing on frailty and quality of life with a sufficient sample are needed.

**Electronic supplementary material:**

The online version of this article (doi:10.1186/s12877-017-0569-2) contains supplementary material, which is available to authorized users.

## Background

Gastric cancer constitutes a major health problem worldwide and is the second most common cause of cancer death [1]. Surgical resection is the main treatment for gastric cancer. Several studies have pointed out that old and young patients carry potential differences in surgery [2, 3]. Older gastric-cancer patients who undergo gastrointestinal surgery may encounter more adverse postoperative outcomes than younger patients [4]. Thus, the need exists to assess the risk of gastrointestinal surgery, especially in older gastric cancer patients.

The prevalence of frailty increases with aging [5]. Frailty is defined as a clinically recognisable state of older adults with increased vulnerability, resulting from age-associated decline in physiological reserve and function across multiple organ systems [5, 6]. Frailty assessment may be a very useful tool for preoperative risk assessment in gastric cancer patients. By assessing frailty, patients can be assigned to undergo either a more tailored individual approach or a standard treatment [5]. Sarcopenia is a syndrome characterised by progressive and generalised loss of skeletal muscle mass and strength [7] and is an important geriatric syndrome closely related to frailty syndrome [8–10]. Increasing evidence shows that frailty or sarcopenia is related to the risk of adverse postoperative outcomes, including morbidity, institutionalisation, prolonged length of hospitalisation, and mortality [2, 9, 11]. Therefore, assessment of frailty and sarcopenia is necessary for older gastric-cancer patients potentially undergoing surgery [5, 12–14].

However, studies concerning the benefit of assessing frailty and sarcopenia in older patients with gastric cancer undergoing gastric surgery are scarce, and the conclusions are inconsistent [2, 9, 11, 15]. Therefore, we conducted this systematic review and meta-analysis aiming to examine the impact of frailty or sarcopenia on postoperative outcomes in older patients undergoing elective gastrectomy surgery.

## Methods

### Search strategy

We searched the following electronic databases: (1) MEDLINE (Ovid, 1946 to August 24, 2016); (2) EMBASE (Ovid, 1974 to August 24, 2016). We used medical subject headings (MeSH) or equivalent and text word terms for MEDLINE and adaptations of the search strategy for EMBASE. We also searched the PubMed web version on August 24, 2016 (https://www.ncbi.nlm.nih.gov/pubmed/), and Google website (www.google.com) for unpublished studies on June 1, 2017. As mentioned in the methodology specified under the PRISMA guidelines (www.prisma-statement.org), two researchers (YJS and JHZ), in collaboration with a medical librarian, performed a systematic search. The following keywords were used for the search: gastric cancer, aged, frailty, sarcopenia, geriatric assessment, postoperative complications and postoperative outcomes. The search string is included in detail in the Additional file 1. We tailored searches to individual databases. The search was completed on August 24, 2016, and animal restriction was applied. In addition, the reference lists of the selected articles were also reviewed to identify relevant articles.

### Study selection

Studies were eligible if they reported postoperative outcomes in older patients with gastric cancer in relation to frailty or sarcopenia profile. We included both retrospective and prospective cohort studies, which described clinical trials in which patients, with an average age of 60 years and older, underwent elective gastric surgery for gastric cancer and were categorised into frail or sarcopenic and non-frail or non-sarcopenic groups. In addition, the criteria used to categorise the patients into frail or sarcopenic groups had to be clearly reported, and frailty or sarcopenia had to be determined in a clinical setting. Studies were excluded if they did not examine postoperative outcomes or surgical complications such as wound infection, anastomotic leakage or mortality as endpoints.

The titles and abstracts of the articles were screened by two investigators (YJS and JHZ). Whenever an article was considered relevant, we reviewed the full text. Finally, to identify potentially eligible studies, we also reviewed all the references in lists of the included studies. We resolved disagreements by discussion to reach a consensus with a third review author (BRD).

### Data charting

One reviewer (YJS) extracted the following data from the included studies: first author, study population, study design, sample size, age of the participants (mean age and standard deviation, if reported), year of publication, country and period of enrolment and inclusion and exclusion criteria of the study. Another reviewer (JHZ) independently double-checked this process. We extracted data regarding the targeted endpoints of this review: criteria and prevalence of frailty and sarcopenia and postoperative outcomes in relation to frailty and sarcopenia groups. If the data in the original manuscript was insufficient, the corresponding author was contacted for additional information.

### Critical appraisal

Reviewers YJS and JHZ independently assessed the methodological quality of the included studies by the Methodological Index for Non-Randomized Studies (MINORS). For non-comparative studies, this instrument consists of the following eight items: (1) a clearly stated aim, (2) inclusion of consecutive patients, (3) prospective collection of data, (4) endpoints appropriate to the aim of the study, (5) unbiased assessment of the study endpoint, (6) follow-up period appropriate to the aim of the study, (7) loss to follow-up less than 5% and (8) prospective calculation of the study size. If the information is not reported, an item is scored 0 points; if the information is reported but inadequate, it scores 1 point; if the information is reported and adequate, it scores 2 points. The ideal score is 16 for non-comparative studies. During a consensus meeting, disagreement among the reviewers was discussed with a third reviewer (BRD).

### Statistical analysis

If there was no available data to extract and pool, we described the outcomes in our review. The summary odds ratios (ORs) and corresponding 95% confidence intervals (CIs) of the included studies were used as measures to assess the association of sarcopenia with postoperative complications. We measured heterogeneity by using the chi^2^ test with significance set at *P* < 0.1. The I^2^ is also computed; it is a quantitative measure of inconsistency across studies. The following is a rough guide to interpretation of I^2^: 0% to 30% might not be important; 30% to 60% may represent moderate heterogeneity; 60% to 75% may represent substantial heterogeneity; 75% to 100% represents considerable heterogeneity. Clinically, there is heterogeneity because of the different evaluation methods of sarcopenia and follow-up time. In consideration of the presence of clinical heterogeneity, we used the random-effects model to synthesise all data, regardless of heterogeneity between the pooled studies in statistical order to obtain more conservative results. Publication bias was assessed by visually inspecting the funnel plots and Egger’s and Begg’s tests (*P* < 0.10). Subgroup analysis was conducted according to different designs of included studies. Sensitivity analysis was performed by omitting each study or included studies with lower quality. The STATA version 11.0 (Stata Corp, College Station, TX, USA) was used to perform all of the analyses. If a *P* value was <0.05, it was statistically significant unless otherwise specified.

## Results

### Selected studies

**Fig. 1:**
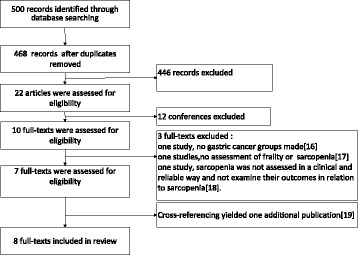
Flowchart of search results and study selection

The search strategy yielded 500 records (Fig. 1), of which 468 after duplications were excluded. After screening the titles and abstracts of the 468 records, 22 articles were assessed for eligibility. Of these articles, 12 of them were conference studies. After reading the remaining 10 full texts, three articles were excluded. One study contained no gastric cancer groups [16]; another study contained no assessment of frailty or sarcopenia [17]; and in one study, sarcopenia was not assessed in a clinical and reliable way, and the outcomes in relation to sarcopenia were not examined [18]. Cross-referencing yielded one additional study [19]. In total, eight studies were included in this systematic review [15, 19–25].

### Study and patient characteristics

In the eight included studies, three studies were prospective observational cohort studies [19, 22, 23], and five studies were retrospective observational cohort studies [15, 20, 21, 24, 25]. The studies were performed from 2005 to 2016 in the Netherlands [15, 20], Japan [21, 22, 25] and China [19, 23, 24]. Five studies described multiple outcomes, including postoperative complications, morbidity, length of stay, a 6-month mortality and in-hospital mortality, hospital costs, 30-day readmission, overall survival and disease-free survival [15, 20–24]. One study focused on surgical site infection [25], and two studies focused on short-term outcomes [19, 23]. The sample size of one study was small (*n* = 99), and the average age of the participants in this study was over 65 years of age [22]. The average age of patients in the remaining seven studies was over 60 years [15, 19–21, 23–25]. The characteristics of the included studies are summarised in Table 1.

**Table 1:** Study and patient characteristics of the included studies

Author, year	Population	Sample	Age, median (range)years	Country	Design	Period	In- and exclusion criteria
Tegels JJ et al. 2014 [20]	gastric adenocarcinoma	180	69.8 (37–88)	Netherlands	retrospective study	1/2005–9/2012	Inclusion- Elective gastric surgery Exclusion - None
Sato T et al. 2016 [21]	gastric cancer	293	66 (33–85)	Japan	retrospective study	5/2011–6/2013	Inclusion - Elective gastric surgery Exclusion - ECOG performance status 3 or 4
Fukuda Y et al., 2016 [22]	gastric cancer	99	> 65	Japan	prospective study	7/2012–1/2015	Inclusion - Elective gastric surgery Exclusion - combined resection
Wang S-L et al. 2016 [23]	ASA grade ≤ III gastric denocarcinoma	255	65.14 (10.81)	China	prospective study	8/2014–3/2015	Inclusion - Elective gastric surgery Exclusion - unresectable
Tegels JJ et al., 2015 [15]	gastric adenocarcinoma	149	69.6 (37–88)	Netherlands	retrospective study	1/2005–9/2012	Inclusion - Elective gastric surgery Exclusion - None
Zhuang CL et al., 2016 [24]	gastric cancer	937	64.0 (median15.0)	China	retrospective study	12/2008–4/2013	Inclusion - Elective gastric surgery Exclusion - None
Chen FF et al., 2016 [19]	undergoing TG with D2 lymphadenectomy for gastric cancer	158	66.9 ± 8.7	China	prospective study	8/2014–2/2016	Inclusion - histologically proven gastric adenocarcinoma -ASA grade of III or less Exclusion - unresectable
Nishigori T et al., 2016 [25]	gastric cancer	157	>60(average age)	Japan	retrospective study	3/2006–10/2014	Inclusion –LTG Exclusion -None

Legend: *SD* standard deviation, *NR* not reported. *LTG* laparoscopic total gastrectomy, *ECOG* Eastern Cooperative Oncology Group

### Quality assessment

The quality assessments of the eight included articles [15, 19–25] are summarised in Table 2. Scores ranged from 13 to 16 with a median value of 14. Every study had collected data retrospectively or prospectively and had included patients consecutively. The follow-up period was appropriate in every study, with a loss to follow-up of less than 5% for all of the studies. Endpoints appropriate to the aim of the study and the prospective calculation of the study endpoint were not reported or reported but inadequate in some of the studies. Furthermore, the unbiased assessment for the study endpoint was not always clear.

**Table 2:** Results of the MINORS quality assessment

Study, Author, year	Clearly stated aim	Inclusion of consecutive patients	Consecutive patients Prospective collection of data	Endpoints appropriate to the aim of the study	Unbiased assessment of the study endpoint	Follow-up period appropriate to the aim of the study	Loss to follow-up <5%	Prospective calculation of the study size	Total
Tegels JJ et al., 2014 [20]	2	2	2	2	0	2	2	1	13
Sato T et al., 2016 [21]	1	2	2	1	2	2	2	1	13
Fukuda Y et al.,2016 [22]	2	2	2	2	1	2	2	1	14
Wang S-L et al., 2016 [23]	2	2	2	2	2	2	2	2	16
Tegels JJ et al., 2015 [15]	2	2	2	2	2	2	2	0	14
Zhuang CL et al.,2016 [24]	2	2	2	2	2	2	2	2	16
Chen FF et al., 2016 [19]	2	2	2	2	2	2	2	2	16
Nishigori T et al., 2016 [25]	1	2	2	1	2	2	2	1	13

Legend: 0 = not reported; 1 = reported but inadequate; 2 = reported and adequate

### Frailty and sarcopenia criteria, groups and prevalence

Only one study described frailty; the remaining seven studies described sarcopenia. One study performed the Groningen Frailty Indicator (GFI) to identify frailty [20]. The prevalence of frailty was 23.62% in the included study.

Two studies used the algorithm of the European Working Group on Sarcopenia in Older Persons (EWGSOP) [15, 22]; another two studies used the Asian Working Group for Sarcopenia (AWGS) and EWGSOP [19, 23]. Patients were considered to have sarcopenia if they met a value that was calculated by the sum of low muscle mass plus low muscle strength and/or low physical performance; then the patients were divided into sarcopenia/non-sarcopenia patients. Although these studies used EWGSOP and AWGS, the cut-off value is different (as shown in Table 3). In addition, one study determined sarcopenia by scoring hand-grip strength [21]. Two studies (Zhuang, CL et al. [24] and Nishigori, T et al. [25]) determined sarcopenia by Assessment Skeletal Muscle Mass, but the cut-off value was different between them, as shown in Table 3. The subjects in our included studies were hospitalised patients with a mean age over 60 years old, and the prevalence of sarcopenia ranged from 12.5% [23] to 57.7% [15].

**Table 3:** Sarcopenia criteria, groups and prevalence

Author, year	Sarcopenia criteria			Sarcopenia groups	prevalence (n,%)
Sato T et al. 2016 [21] (*n* = 293)	Hand grip strength	High HGS ≥ GSL 20% Low HGS<GSL 20%	<27.5 kg in men <16.2 kg in women	High HGS Low HGS	239(81.57%) 54(18.43%)
Fukuda Y et al., 2016 [22] (*n* = 99)	EWGSOP	4-m Gait speed	≤0.8 m/s	Sarcopenic Non-sarcopenic	21(21.21%) 78(78.79%)
		hand grip strength	<30 kg for men <20 kg for women		
		whole-body skeletal muscle mass(BIA)	<8.87 kg/m^2^ for men <6.42 kg/m^2^ for women		
Wang S-L et al., 2016 [23] (*n* = 255)	EWGSOP AWGS	L3 skeletal muscle index (SMI)	<36.0 cm^2^/m^2^ in men <29.0 cm^2^/m^2^ in women	Sarcopenic Non-sarcopenic	32(12.50%) 223(87.50%)
		hand grip strength	<26 kg for men <18 kg for women		
		6-m gait speed	≤0.8 m/s		
Tegels JJW et al., 2015 [15] (*n* = 149)	EWGSOP	L3 skeletal muscle index (SMI)	43 cm^2^/m^2^ for males with BMI < 25.0 cm^2^/m^2^ 53 cm^2^/m^2^ for males with BMI ≥25.0 cm^2^/m^2^ in females threshold for sarcopenia was 41 cm^2^/m^2^	Sarcopenic Non-sarcopenic	86(57.70%) 63(42.30%)
Zhuang CL et al., 2016 [24] (*n* = 937)	Skeletal Muscle Mass	L3 skeletal muscle index (A cross-sectional CT image)	34.9 cm^2^/m^2^ for women 40.8 cm2/m2 for men	Sarcopenic Non-sarcopenic	389(41.50%) 548(58.50%)
Chen FF et al., 2016 [19] (*n* = 158)	EWGSOP and AWGS	L3 skeletal muscle index (SMI)	<34.9 cm^2^/m^2^ for women <40.8 cm^2^/m^2^ for men	Sarcopenic Non-sarcopenic	39(24.70%) 119(75.30%)
		hand grip strength	<26 kg for men <18 kg for women		
		6-musual gait speed	<0.8 m/s		
Nishigori T et al., 2016 [25] (*n* = 157)	Skeletal muscle mass	L3 skeletal muscle index (A cross-sectional CT image)	≤52.4cm^2^/m^2^ for men ≤38.5cm^2^/m^2^ for women	sarcopenic nonobesity sarcopenic obesity nonsarcopenic nonobesity nonsarcopenic obesity	52(33.12%) 45(28.66%) 32(20.38%) 28(17.83%)

Legend: *EWGSOP* the European Working Group on Sarcopenia, *AWGS* the Asian Working Group for Sarcopenia, *BIA* bioimpedance analysis, *HGS* hand grip strength, *GSL* gender-specific lowest 20th percentile, *SMI* skeletal muscle index, *BIA* bioimpedance analysis, *ASM* appendicular skeletal muscle mass

### Frailty or sarcopenia and postoperative outcomes

The outcomes reported in all included studies in relation to frailty or sarcopenia were in-hospital mortality [20], postoperative complications [19, 21–25], serious adverse events [20, 22, 24], hospital costs [23], overall survival [24], disease-free survival [24] and surgical site infection [25]. The postoperative complications of the studies were classified into different severity grades by using a well-described classification system (Clavien–Dindo, 2004) [26]. The classification systems used are summarised in Table 4.

**Table 4:** Severity grading classification systems of surgical complications

	Clavien-Dindo, (2004) [26]
Grade I	Any deviation from the normalpostoperative course without the need for pharmacological treatment or surgical,endoscopic, and radiological interventions. Allowed therapeutic regimens are drugs such as antiemetics, antipyrectics, analgetics, diuretics, electrolytes, and physiotherapy. This grade also includes wound infections opened at the bedside
Grade II	Requiring pharmacological treatment with drugs other than such allowed for grade I complications. Blood transfusions and parenteral nutrition are also included.
Grade III	Requiring surgical, endoscopic, or radiological intervention. IIIa: Intervention not under general anaesthesia. IIIb: Intervention under general anaesthesia.
Grade IV	Life-threatening complication (including CNS ^a^ complications) requiring IC/ICU management IVa: Single organ dysfunction (including dialysis) IVb: Multiorgan dysfunction
Grade V	Death of a patient
Grade VI	/
Suffix ‘d’	If the patient suffers from a complication at the time of discharge, the suffix ‘d’ (for ‘disability’) is added to the respective grade of complication. This label indicates the need for a follow-up to fully evaluate the complication.

Legend: *CNS* central nervous system, *IC* intermediate care, *ICU* intensive care unit

^a^Brain hemorrhage, ischemic stroke, subarachnoidal bleeding, but excluding transient ischemic attacks

One study (Tegels, JJ et al.) [20] used GFI ≥3 to define frailty, and the results show a significant relationship between frailty and surgical mortality in gastric cancer (OR 3.96; 95% CI: 1.12 to 14.09, *P* = 0.03). This study also explored the relationship between frailty and serious adverse events, length of stay, and 6-month mortality. In this study, frailty was associated with increased risk of serious adverse events (defined as Clavien–Dindo grade 3a or over); however, frailty did not correlate with either increased 6-month mortality or increased length of stay.

**Fig. 2:**
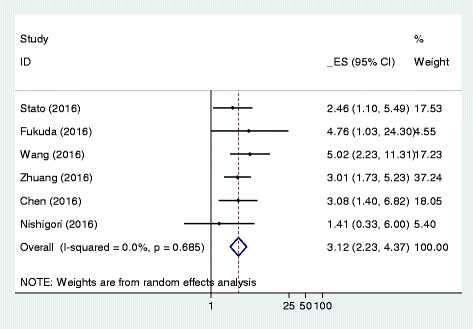
Forest plot of the odds ratios for the association between sarcopenia and postoperative complications of gastric cancer

**Fig. 3:**
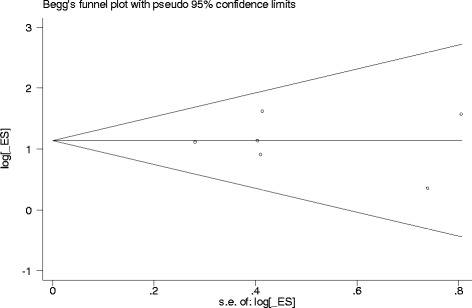
Funnel plot of sarcopenia and postoperative complications of gastric cancer

Six studies reported the association between sarcopenia and postoperative complications. We calculated the summary OR values using random-effects models; the pooled OR of gastric cancer from the combination of included studies was 3.12 (95% CI: 2.23–4.37) for postoperative outcome (Fig. 2). This indicated that sarcopenia was an independent risk factor for severe postoperative complications. No significant heterogeneity was observed across these pooled studies (Chi^2^ = 3.10, df = 5, I^2^ = 0%, *P* = 0.685) (Fig. 3) [19, 21–25].

### Publication bias

Begg’s and Egger’s tests were performed to assess the publication bias in these studies. The results did not show any statistical significance for publication bias (Begg’s test: *p* = 0.851; Egger’s test: *p* = 0.840). Furthermore, the shape of the funnel plot did not reveal any evidence of obvious asymmetry (Fig. 3).

### Subgroup and sensitivity analysis

We conducted the subgroup analysis according to the study design (prospective and retrospective studies); the statistical significance was not changed by this subgroup analysis (prospective studies: OR 3.12, 95% CI (2.23, 4.37), *I* = 0%, *P* value for heterogeneity 0.684; and retrospective studies: OR 2.65, 95% CI (1.72, 4.09), *I* = 0%, *P* value for heterogeneity was 0.617). We performed sensitivity analysis by omitting every single study and two low quality studies of the included studies [15, 20] (quality score); the statistical significance of the results were still not changed (data not shown).

## Discussion

To the best of our knowledge, this is the first meta-analysis to explore systematically the impact of frailty and sarcopenia in predicting outcomes among older patients undergoing gastrectomy surgery. Although only one included study focused on frailty and gastrectomy surgery in this review, frailty was still a statistically significant factor for predicting surgical mortality in the older patient (OR 3.96, 95% CI 1.12–14.09). There was also a significant relationship between sarcopenia and postoperative complications (OR 3.12, 95% CI: 2.23–4.37) in older people. Our study indicated that assessing frailty and sarcopenia is important among older patients undergoing gastrectomy surgery.

The prevalence of sarcopenia ranged from 5% to 13% for older people aged 60 to 70 years and ranged from 11% to 50% for people aged 80 and older [27, 28]. The prevalence of sarcopenia among patients with gastric cancer has been reported to be as high as 38% [9]. In this systematic review, the prevalence of sarcopenia ranged from 12.5% [23] to 57.7% [15]. The difference in this prevalence can be explained by patients with gastric cancer being at a particularly high risk of sarcopenia and the different diagnostic criteria used in different studies. Even though these criteria had been established long ago, uniform criteria are still not established. Thus, different cut-off points for muscle mass and muscle strength were used in different studies (Table 3). In the present study, the EWGSOP and AWGS algorithm, hand grip strength (kg) and skeletal muscle mass were considered suitable methods for the diagnosis of sarcopenia. This may be for reasons of clinical heterogeneity.

Previous studies showed that the state of frailty and sarcopenia in the preoperative period were related to the occurrence of adverse postoperative outcomes, including morbidity, mortality, institutionalisation and prolonged length of hospitalisation [2, 9, 11]. Recently, Doris Wagner and colleagues [9] performed a systematic review regarding the role of frailty and sarcopenia in predicting outcomes among patients undergoing gastrointestinal surgery. However, this review included not only gastrectomy surgery, but also gastroesophageal surgery, colorectal surgery and hepatopancreaticobiliary surgery. Because of the high level of heterogeneity, the authors did not do meta-analysis. Furthermore, they limited their search date to January 2000 to March 2015 and only considered studies published in English. Therefore, our systematic review and meta-analysis focuses on gastrectomy surgery to reduce some of the clinical heterogeneity, giving us an opportunity to conduct the meta-analysis in this review. To the best of our knowledge, there was no systematic review that reported the role of sarcopenia in predicting morbidity and mortality of patients undergoing gastric surgery. Another systematic review conducted by Kathleen Fagard and colleagues [29] suggested that frailty is associated with a greater risk of postoperative adverse outcomes in patients with colorectal cancer. Our study found frailty had a predictive capacity for in-hospital mortality [20] and serious adverse events [20], but not for 6-month mortality and length of stay [20]. Sarcopenia is associated with mortality [21], postoperative complications [19, 21–24], hospital costs [23], postoperative hospital stay [23], overall survival [24] and disease-free survival [24].

Our results must be interpreted with caution due to the following limitations. First, although we conducted subgroup and sensitivity analyses according to the design and quality of the included studies, we did not perform the subgroup analyses according to the different sarcopenia criteria. All these factors may cause heterogeneity. Second, even frailty and sarcopenia are common in the elderly, the included criteria of our review was based on the average age of 60 years or more. However, two included studies might have included some participants younger than 60 years, which may cause some bias. Third, whether our results can be applied to Western populations remains unknown because all the included studies were from Asian countries. Fourth, none of the included studies reported quality of life as a primary outcome. Therefore, future studies should focus on more patient-centred outcomes such as quality of life. Fifth, only one study focused on the frailty assessment in predicting postoperative complications in gastric cancer surgery. However, frailty is a very important geriatric syndrome in older people. Therefore, future studies focusing on frailty and postoperative complications in gastric cancer are needed.

## Conclusion

Sarcopenia and frailty seem to have a significant impact on the occurrence of adverse postoperative outcomes. Thus, it is important to define whether a patient with gastric cancer has sarcopenia and is frail in the perioperative period. Further well-designed, prospective, cohort studies focusing on frailty and quality of life with sufficient samples are needed.

## Additional file

Additional file 1: MEDLINE search strategy. (DOCX 12 kb)

## Abbreviations

ASM: appendicular skeletal muscle mass
BIA: bioimpedance analysis
ECOG: Eastern cooperative oncology group
EWGSOP: European Working Group on Sarcopenia in Older Persons
GFI: Groningen frailty indicator
GSL: gender-specific lowest 20th percentile
HGS: hand grip strength
HR: hazards ratio
LTG: laparoscopic total gastrectomy
NR: not reported
OR: odds ratio
SD: standard deviation
SMI: skeletal muscle index
