# Supplementary material for: The impact of frailty and sarcopenia on postoperative outcomes in older patients undergoing gastrectomy surgery: a systematic review and meta-analysis
Source: BMC Geriatr. 2017 Aug 21;17:188. doi: 10.1186/s12877-017-0569-2 (PMC5563908; doi:10.1186/s12877-017-0569-2)
Supplement: Additional file 1: — MEDLINE search strategy. (DOCX 12 kb) [file 12877_2017_569_MOESM1_ESM.docx]

**Appendix A.**

**MEDLINE search strategy**

1. exp Sarcopenia/ or exp Muscular Atrophy/

2. ("Muscular Atrophy" or Sarcopenia or Sarcopenias or Sarcopenic or presarcopenia or Samopenia).af.

3. Exp Frail Elderly/

4.frail$.af.

5.(carcin$ or cancer$ or neoplas$ or tumour$ or tumor$ or cyst$ or growth$ or adenocarcin$ or malig$).mp.

6.(Digest$ or Gastr$ or epigastr$ or stomach$).mp.

7. exp Stomach Neoplasms/

8. gastric cancer.mp.

9. gastric neoplasm*.mp.

10. Stomach cancer.mp.

11. Stomach neoplasm*.mp.

12. stomachus cancer.mp.

13. stomachus neoplasm*.mp.

14. gaster cancer.mp.

15. gaster neoplasm*.mp.

16.1 OR 2

17. 3 OR 4

18. 5 AND 6

19. 7-15/ OR

20. 18 OR 19

21. 16 OR 17

22.20 AND 21
